# Chromothripsis-like chromosomal rearrangements induced by ionizing radiation using proton microbeam irradiation system

**DOI:** 10.18632/oncotarget.7186

**Published:** 2016-02-04

**Authors:** Maki Morishita, Tomoki Muramatsu, Yumiko Suto, Momoki Hirai, Teruaki Konishi, Shin Hayashi, Daichi Shigemizu, Tatsuhiko Tsunoda, Keiji Moriyama, Johji Inazawa

**Affiliations:** ^1^ Department of Molecular Cytogenetics, Medical Research Institute, Tokyo Medical and Dental University, Tokyo, Japan; ^2^ Department of Maxillofacial Orthognathics, Graduate School of Medical and Dental Sciences, Tokyo Medical and Dental University, Tokyo, Japan; ^3^ Research Fellow of the Japan Society for the Promotion of Science, Chiyoda-ku, Tokyo, Japan; ^4^ Biodosimetry Research Team, Research Center for Radiation Emergency Medicine, National Institute of Radiological Sciences, Inage-ku, Chiba-shi, Chiba, Japan; ^5^ Research Development and Support Center, National Institute of Radiological Sciences, Inage-ku, Chiba-shi, Chiba, Japan; ^6^ Department of Medical Science Mathematics, Medical Research Institute, Tokyo Medical and Dental University, Tokyo, Japan; ^7^ Laboratory for Medical Science Mathematics, RIKEN Center for Integrative Medical Sciences, Tsurumi, Yokohama, Japan; ^8^ Bioresource Research Center, Tokyo Medical and Dental University, Bunkyo-ku, Tokyo, Japan

**Keywords:** cancer, chromothripsis, microbeam, irradiation, DNA damage

## Abstract

Chromothripsis is the massive but highly localized chromosomal rearrangement in response to a one-step catastrophic event, rather than an accumulation of a series of subsequent and random alterations. Chromothripsis occurs commonly in various human cancers and is thought to be associated with increased malignancy and carcinogenesis. However, the causes and consequences of chromothripsis remain unclear. Therefore, to identify the mechanism underlying the generation of chromothripsis, we investigated whether chromothripsis could be artificially induced by ionizing radiation. We first elicited DNA double-strand breaks in an oral squamous cell carcinoma cell line HOC313-P and its highly metastatic subline HOC313-LM, using Single Particle Irradiation system to Cell (SPICE), a focused vertical microbeam system designed to irradiate a spot within the nuclei of adhesive cells, and then established irradiated monoclonal sublines from them, respectively. SNP array analysis detected a number of chromosomal copy number alterations (CNAs) in these sublines, and one HOC313-LM-derived monoclonal subline irradiated with 200 protons by the microbeam displayed multiple CNAs involved locally in chromosome 7. Multi-color FISH showed a complex translocation of chromosome 7 involving chromosomes 11 and 12. Furthermore, whole genome sequencing analysis revealed multiple *de novo* complex chromosomal rearrangements localized in chromosomes 2, 5, 7, and 20, resembling chromothripsis. These findings suggested that localized ionizing irradiation within the nucleus may induce chromothripsis-like complex chromosomal alterations via local DNA damage in the nucleus.

## INTRODUCTION

Carcinogenesis has been generally explained by the multistep theory of carcinogenesis, in which tumors form by the accumulation of somatic mutations over a long period of time [1, 2]. However, chromothripsis has recently been proposed as a new and completely different process of carcinogenesis. Chromothripsis is a one-step catastrophic cellular event in cancer, in which chromosomes undergo localized massive copy number alterations (CNAs) and rearrangements [3, 4]. Chromothripsis has been reported to occur in almost all cancer types with a prevalence of 2-3% [5], suggesting that it may be a common factor of carcinogenesis in every organs. High frequency of chromothripsis is thought to be due to chromosomal instability caused by *TP53* mutations and impairment of DNA repair machineries. Additionally, because chromothripsis causes chromosomal alterations and/or mutations, it is thought to be closely associated with the development of high-grade cancer [6, 7]. Chromothripsis is defined by the following three features: (1) localized rearrangements in limited areas of one or several chromosomes, (2) copy number states that suddenly oscillate between areas of normal heterozygosity and loss of heterozygosity and (3) multiple chromosome fragments rearranged in random orientation and order [8, 9]. Although phenomenons of chromothripsis have been reported in many studies, the mechanism by which chromothripsis occurs remains unclear. One mechanism that has been proposed is the shattering of DNA into pieces within a micronucleus which is produced as a result of cell division defects and errors in DNA replication or repair; the micronucleus is then incorporated into a single chromatid [10, 11]. However, how chromosome fragmentation is induced and what factors are associated with chromosome shattering remain to be elucidated.

DNA double strand breaks (DSBs) and the formation of micronuclei by DNA-damaging factors, such as oxygen free-radicals, chemical compounds, and ionizing radiation, are thought to induce chromothripsis [4]. For example, treatment with nocodazole, a microtubule polymerization inhibitor, has been reported to form micronuclei and to induce chromothripsis within these micronuclei [10, 11]. Moreover, it has also been suggested that ionizing radiation may induce chromothripsis via DNA damage [3]. Ionizing radiation is widely used for medical treatment, such as X-rays, CT, and radiation therapy for cancer patients. On the other hand, it is well known that some types of cancer, such as skin cancer and thyroid cancer, are generated by ionizing radiation [12–14]. Many studies have demonstrated that ionizing radiation gives rise to chromosomal abnormalities, such as amplification, deletion, inversion, and translocation [13, 15].

Here, we established irradiated monoclonal sublines from two types of oral squamous cell carcinoma (OSCC) cell lines originating from an identical OSCC cell line, using the Single Particle Irradiation system (SPICE), a focused vertical microbeam system designed to irradiate the nuclei of adhesive cells [16], to investigate the mechanism by which chromothripsis occurs. SPICE is able to precisely target a proton beam to only the nucleus, efficiently inducing local DSBs in some chromosomes. Then, we analyzed the genomic status in established sublines by SNP array, multi-color FISH (M-FISH), and whole genome sequencing (WGS), and detected chromothripsis-like complex chromosomal alterations in one subline. Our findings suggested that ionizing radiation may produce chromothripsis via DNA damage.

## RESULTS

### Establishment of irradiated monoclonal sublines

**Figure 1:**
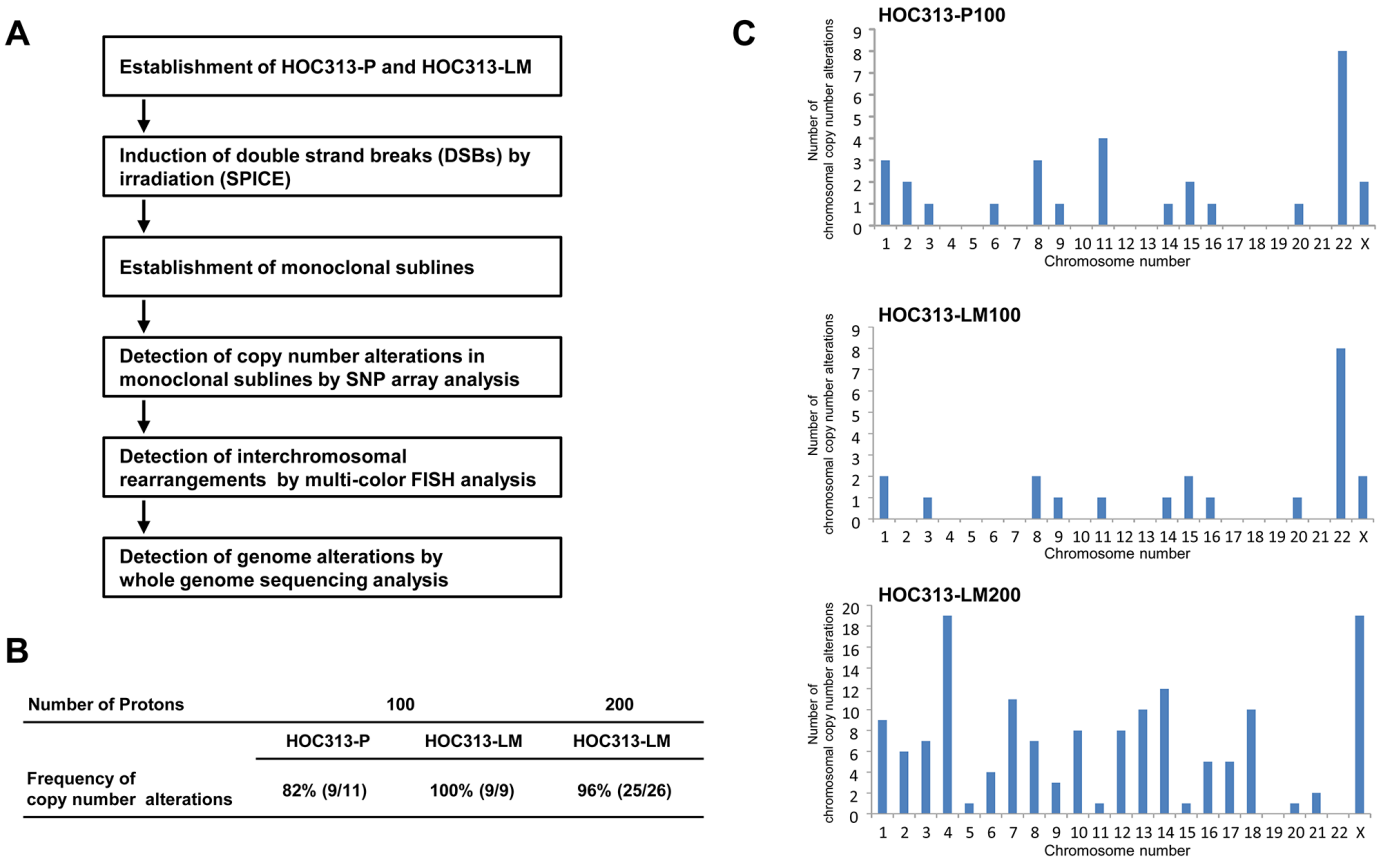
Establishment of the irradiated monoclonal sublines from the oral squamous cell carcinoma cell lines, HOC313-P and HOC313-LM, and the detection of radiation-induced chromosomal CNAs by SNP array analysis **A.** Strategy of the establishment of the irradiated monoclonal sublines by microbeam irradiation using SPICE, and the detection of chromosomal alterations by several genome analysis methods. **B.** The detection frequency of chromosomal CNAs by SNP analysis. HOC313-P and HOC313-LM were irradiated with 100 or 200 protons at one cell nucleus. Percentage indicates the frequency of the monoclonal sublines harboring CNAs. **C.** The frequency of CNAs in each chromosome in HOC313-P100 (upper), -LM100 (middle), and -LM200 (bottom). The abscissa indicates chromosome number and the ordinate indicates the summation of chromosomal CNAs detected by SNP array analysis in the monoclonal sublines.

To obtain accurate genetic information for identifying the effects of irradiation in cancer cells, we first established monoclonal sublines from HOC313-P, an OSCC cell line, and HOC313-LM, a highly metastatic cell line derived from HOC313-P [17] (Figure 1A). We next confirmed the monoclonal sublines' resistance to irradiation and formation of micronuclei, an index of chromothripsis. The resistance to irradiation in HOC313-LM was higher than HOC313-P (Supplementary Figure S1), and formation of micronuclei was observed in irradiated HOC313-LM cells (Supplementary Figure S2). Because the formation of micronuclei was induced by irradiation, we concluded that the HOC313-P and -LM cell lines were suitable for chromothripsis analysis. To induce DSBs in HOC313-P and HOC313-LM, we irradiated these cell lines using the SPICE irradiation system. These cell lines were irradiated with 100 or 200 protons as described in Materials and Methods. The irradiated area was 3.14 μm^2^ within the nucleus (Supplementary Figure S3), and the absorbed dose (Gy) for the irradiated area was 55 Gy or 110 Gy for 100 or 200 protons, respectively. After irradiation with 200 protons, all HOC313-P-derived monoclonal sublines experienced cell death, whereas 26 HOC313-LM-derived monoclonal sublines were successfully established; this may be due to the tendency of higher radiation resistance of HOC313-LM compared with HOC313-P (Supplementary Figure S1). Each established HOC313-P- or HOC313-LM-derived monoclonal subline was named HOC313-P100 (#1 - #11), LM100 (#1 - #9), and LM200 (#1 - #26), with 100 or 200 indicating the number of protons the cells had been irradiated with.

### CNAs induced by DSBs in SPICE-irradiated cells

To investigate whether CNAs are induced by irradiation, we analyzed DNA copy number states using SNP array in each irradiated monoclonal subline. We used the genomic segmentation algorithm [18] and determined the appropriate threshold values for detecting CNAs as <1.8 copies (deletion) and >2.6 copies (amplification). Using these threshold values, we were able to detect chromothripsis in the thyroid anaplastic carcinoma cell line 8505C, which has been reported to have chromothripsis at the short arm of chromosome 9 (Supplementary Figure S4). Thus, we used these threshold values for SNP array statistical processing, resulting in a ratio of monoclonal sublines with *de novo* CNAs to total established monoclonal sublines of 82% (9/11), 100% (9/9) and 96% (25/26) for the HOC313-P100, -LM100, and -LM200 sublines, respectively (Figure 1B). When the HOC313-P and -LM monoclonal sublines were irradiated with 100 protons, the chromosomes involved in the irradiation-induced CNAs were similar in both the HOC313-P100 and HOC313-LM100 sublines (Figure 1C). Moreover, irradiation with 200 protons induced CNAs in almost all chromosomes, other than chromosomes 19 and 22, and was most notable in chromosomes 1, 4, 14, 22, and X (Figure 1C). Four (out of 14) chromosomal alterations, which occurred at chromosome 1, were deletions near the common fragile site FRA1H: 1q42.1 (Supplementary Table S1). Fragile sites exhibit an increased frequency in occurrence of gaps or breaks when exposed to DNA damage, such as irradiation [19]; hence, the deletions observed in the present study at fragile sites and/or their vicinity were likely to have been acquired by irradiation. Furthermore, in FRA16D (16p23), which is known to be a common fragile site, high-frequency loss was detected by SNP array (Supplementary Figure S5). *WWOX*, a tumor suppressor gene, is located in this region and is observed as a frequent loss due to heterozygosity or homozygous deletions in several human cancers [20]. These results suggested that focal irradiation by SPICE induces CNAs in almost all chromosomes, including fragile sites, similar to that by conventional whole-nuclei irradiation.

### Multiple CNAs detected by SNP array and M-FISH

**Figure 2:**
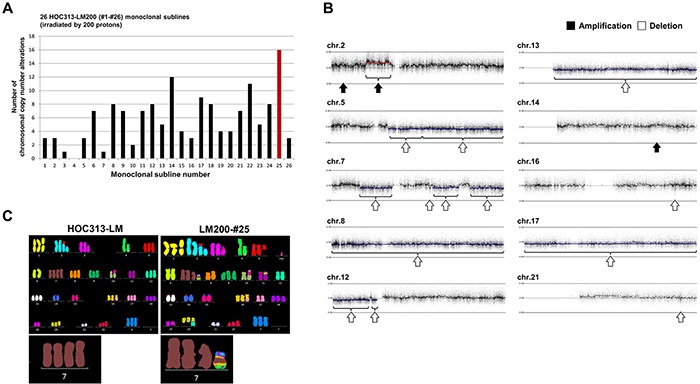
CNAs were induced locally in chromosome 7, including translocation of chromosomes 11 and 12 in LM200-#25 **A.** The total number of chromosomal CNAs induced by irradiation in each of the 26 HOC313-LM200 monoclonal sublines. Red bar indicates the number of CNAs in HOC313-LM200-#25. **B.** Results of SNP array profiles in LM200-#25. (black arrows: amplification. white arrows: deletion.) **C.** M-FISH analysis shows the translocation of chromosome 11 and 12 in chromosome 7 in LM200-#25. (Left image: HOC313-LM [before irradiation] Right image: LM200-#25 [after irradiation]) Enlarged chromosomes 7 are shown below.

To investigate the effect of irradiation, we analyzed the total number of *de novo* CNAs that occurred in each monoclonal subline. Almost all monoclonal sublines had several CNAs, and LM200-#25 showed 16 *de novo* CNAs (Figure 2A, Table 1). These alterations included whole-chromosome deletions in chromosomes 8, 13, and 17, and interestingly, multiple deletions involved in different regions of chromosome 7 (Figure 2B). These findings suggested that cryptic chromosomal alterations, including a chromothripsis-like phenotype, may be involved in chromosome 7 in LM200-#25. Thus, we next performed M-FISH analysis in HOC313-LM and LM200-#25 (Figure 2C). While tetrasomy chromosome 7 was identified in HOC313-LM, LM200-#25 had three normal chromosome 7 together with a short derivative of chromosome 7 involved in chromosomes 11 and 12. Moreover, deletions of chromosomes 8, 13, and 17, as detected by SNP array analysis, were also observed by M-FISH, suggesting that our threshold values for SNP array were reasonable for detection of CNAs.

**Table 1:** Summary of chromosomal CNAs in LM200-#25 by SNP array analysis

Chromosome Number	Copy Number	Position	Start Position	End Position	Length (bps)
2	Amp	2p24.3	16,112,059	16,374,530	262,471
2	Amp	2p16.3 - 2p11.2	48,874,201	83,960,470	35,086,269
5	Del	5q12.1 - 5q15	60,181,452	95,997,250	35,815,798
5	Del	5q15 - 5q35.3	96,133,485	180,693,128	84,559,643
7	Del	7p15.2 - 7p11.2	26,267,811	56,070,567	29,802,756
7	Del	7q21.13 - 7q21.2	90,400,492	91,667,757	1,267,265
7	Del	7q21.3 - 7q31.31	93,403,513	117,737,887	24,334,374
7	Del	7q32.1 - 7q36.3	127,700,973	159,119,487	31,418,514
8	Del	8p23.3 - 8q24.3	164,984	146,293,415	146,128,431
12	Del	12p13.33 - 12p11.22	191,619	28,788,116	28,596,497
12	Del	12p11.22 - 12p11.1	30,194,870	34,823,250	4,628,380
13	Del	13q11 - 13q34	19,058,717	115,103,530	96,044,813
14	Amp	14q31.1	82,396,465	82,542,289	145,824
16	Del	16q23.1	78,925,534	79,054,884	129,350
17	Del	17p13.3 - 17q25.3	8,547	81,051,008	81,042,461
21	Del	21q22.3	43,557,092	43,634,574	77,482

* Amp; amplification, Del; deletion

### Chromothripsis-like complex chromosomal rearrangements occurred after SPICE-irradiation

**Figure 3:**
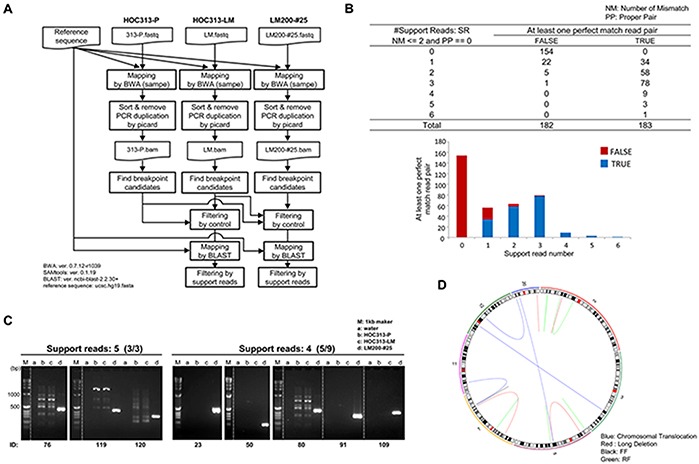
Whole genome sequencing analysis identified microbeam irradiation-induced *de novo* chromosomal rearrangements **A.** Identification scheme of *de novo* rearrangements in LM200-#25. Detailed information is described in Materials and Methods. **B.** The summary of support read number after filtering. Candidates of *de novo* rearrangements supported by ≤ 3 read pairs were regarded as true. NM: number of mismatches, PP: proper pair. **C.** Validation of *de novo* rearrangement candidates by PCR. M: 1kb marker, a: control, b: HOC313-P, c: HOC313-LM, d: LM200-#25. **D.** CIRCOS plots of LM200-#25, demonstrating occurrence of *de novo* rearrangements in chromosomes 2, 5, 7, and 20 at high frequency. Each line color represents the type of rearrangement. Blue: chromosomal translocation, Red: long deletion, Pink: forward and forward (FF), and Black: reverse and forward (RF).

To obtain detailed genomic information in LM200-#25, we performed WGS analyses in HOC313-P, -LM, and LM200-#25. *De novo* rearrangements in LM200-#25 were predicted by clustering of inconsistent read pairs (Figure 3A, see Materials and Methods). Common rearrangements observed between HOC313-P and -LM were filtered out, and 91 rearrangements supported by at least three read pairs were extracted as candidate breakpoints (Figure 3B and Supplementary Table S2). These 91 candidate breakpoints were validated by Sanger sequencing of DNA amplified by PCR in HOC313-P, -LM, and LM200-#25. Of them, 14 were identified as *de novo* rearrangements induced by irradiation in LM200-#25 (Figure 3C and Supplementary Figure S6). The remaining 77 were false-positives either obtained from all cell lines or never obtained (Supplementary Figure S6).

The confirmed *de novo* rearrangements were located on chromosomes 2 (3/14), 5 (4/14), 7 (4/14), and 20 (3/14) at high frequency (Figure 3D, Supplementary Figure S7 and Table 2), suggesting that these focal chromosomal rearrangements resembled chromothripsis-like complex chromosome rearrangements. WGS analysis then detected not only the translocation observed by M-FISH analysis (chromosome 11 in chromosome 7 of ID120), but also that, not detected by M-FISH analysis (chromosome 5 and 20 of ID91) (Table 2). Furthermore, it revealed that several breakpoints were located in close proximity: the distance between breakpoint 2 of ID80 and breakpoint 1 of ID91 was 377 bp; the distance between breakpoints 1 of ID119 and ID120 was only 5 bp. While two chromosome copy number gains were detected within chromosome 2p by SNP array, the regions retained heterozygosity, concordant with one of the definitions of chromothripsis [21]. These results suggested that multiple and highly complex chromosomal rearrangements were induced coincidentally in LM200-#25 via a single destructive event of DNA damage by SPICE microbeam irradiation.

**Table 2:** Summary of chromosomal rearrangements by whole genome sequencing analysis in LM200-#25

ID	Break 1 Chromosome	Break 1 Position	Break 2 Chromosome	Break 2 Position	Rearrangement Type	Sequence in Between Breakpoints
23	2	15,742,392	2	16,708,640	RF	CTGAA insertion
29	2	48,937,454	2	81,807,507	RF	C microhomology
30	2	53,106,380	2	53,252,037	LD	AA microhomology
50	3	144,705,944	12	28,795,184	CT	TT microhomology
76	5	6,330,710	5	6,378,473	RF	TG microhomology
80	5	19,854,137	5	**89,613,562**	LD	
91	5	**89,613,939**	20	14,453,942	CT	TGGA microhomology
93	5	95,994,652	5	96,157,380	RF	GAT microhomology
108	7	26,274,055	7	123,601,513	LD	
109	7	51,228,098	7	51,281,802	RF	TT microhomology
119	7	**127,757,875**	7	**127,820,864**	FF	TTT microhomology
120	7	**127,757,880**	11	23,404,604	CT	
143	12	53,845,481	20	5,707,700	CT	G microhomology
167	20	57,562,329	20	57,708,236	LD	GCTCTGGTCCTGCATGACGTCCGTAGGATCACTT insertion

*Bold numbers indicate that several breakpoints were located in close proximity

CT chromosomal translocation

LD long deletion

FF forward and forward

RF reverse and forward

### *De novo* rearrangement junction pattern was non-homologous end joining (NHEJ)

It has previously been reported that the mechanism of DSB repair in chromothripsis involves NHEJ or alternative non-homologous end joining (Alt-NHEJ) [22, 23]. In order to investigate the breakpoints in more detail, we performed Sanger sequencing of 14 breakpoints using DNA amplified by PCR in LM200-#25 (Table 2). As a result, microhomology of up to four base pairs was observed at nine junction points: a blunt fusion pattern (NHEJ) at three, and insertion at two (Table 2). While the insertion sequence in ID23 was short (5 bp) and its origin was unknown, insertion sequence in ID167 was 33 bp sequence derived from the sequence 79 bp upstream breakpoint 2 in reverse orientation. Taken together, we concluded that most of the chromosome fragments, shattered by irradiation, were joined together by NHEJ or Alt-NHEJ.

## DISCUSSION

In the present study, we observed that chromothripsis-like complex rearrangements were induced by microbeam irradiation using SPICE in cancer cell nuclei of an OSCC cell line, HOC313-LM200-#25. These findings are the first report suggesting that ionizing radiation may contribute in part to the occurrence of multiple chromosomal rearrangements acquired in a one-off event, chromothripsis. The number of chromothriptic rearrangements detected in LM200-#25 was less than that reported in previous studies [3, 24, 25], but the features and essential pattern of DNA rearrangements were similar to those in chromothripsis, in other words, showing chromothripsis-like complex chromosomal rearrangements as described in previous reports [26, 27].

**Figure 4:**
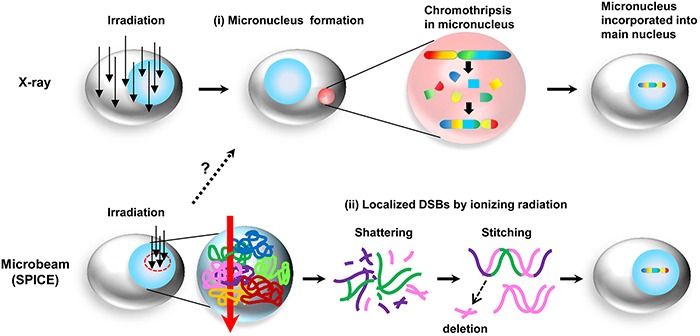
Hypothetic schematic representation of the mechanisms of chromothripsis induced by ionizing radiation **(i)** Chromothripsis occurs via micronuclei generated by ionizing radiation. When the entire cell is irradiated, for example, X-ray, the radiation damage induces formation of micronuclei. After chromothripsis occurs in the micronuclei, these micronuclei are then incorporated into a single chromatid. **(ii)** Chromothripsis may be directly induced by ionizing irradiation. When a portion of the nuclei is irradiated by microbeam (SPICE), multiple chromosomes sitting in the irradiated area are shattered into pieces, which are then rejoined via NHEJ.

The most critical question is whether the detected rearrangements in LM200-#25 depended on a single destructive event by irradiation, or the accumulation of mutations during cell culture. Some deletions in chromosome 7 were detected by SNP array analysis, and M-FISH analysis revealed translocation between chromosomes 7, 11, and 12. In addition, the breakpoints of the rearrangements in chromosome 7 were in close proximity to ID108, ID119, and ID120 (Table 2). A similar phenomenon occurred not only in chromosome 7, but also in chromosomes 2, 5, and 20. Taken together, although the number of rearrangements in LM200-#25 was less than that has been previously reported [3, 24, 25, 28], we believe that the detected chromosomal rearrangements in LM200-#25 may have been due to a single destructive event by focal irradiation using SPICE (Figure 4). In the present study, we performed irradiation using SPICE with 100 or 200 protons within a limited circular-area of approximately 2-μm diameter in the nuclei of OSCC cell lines HOC313-P and -LM, and detected chromothripsis-like complex chromosomal rearrangements in one subline, LM200-#25. However, an optimal radiation dose for consistent induction of chromothripsis has not yet been determined. The microbeam radiation dose and the size and area of the irradiated field may be critical for inducing chromothripsis; but it was difficult to predict the optimal conditions of irradiation to induce chromothripsis in cells. More detailed understanding of the mechanism underlying chromothripsis induction by irradiation may be possible if more optimal conditions could be obtained for inducing one-off catastrophic DNA rearrangements by SPICE. Moreover, there is a possibility that the number of rearrangements in LM200-#25 detected by WGS analysis was less than the actual number of rearrangements. In fact, in some respects, the results by WGS analysis were inconsistent with the results of M-FISH, for example, albeit the detection of chromosome 12 translocation in chromosome 7 by M-FISH in all examined metaphase cells, it was not detected by WGS analysis. One reason for this inconsistency may be due to the difficulty in detecting rearrangements by WGS analysis compared with detecting single nucleotide variants, insertions, and deletions, due to a lack of well-established algorithms in WGS analysis, resulting in false-negatives. In addition, because the sequence length was at most 125 bp, which is too short to map to the reference genome sequence, the detection of rearrangements was difficult within the repeat region. The detection of *de novo* rearrangements by WGS analysis may have been more difficult by using cancer cell lines, which harbor massive native rearrangements in comparison with normal cells.

SNP analysis showed that a high frequency of deletions occurred in each monoclonal subline at 16q23.1, which is upstream of one of the most well-known common fragile sites, 16q23.2 (FRA16D) (Supplementary Figure S5A). Fragile sites are known to be chromosomal structures sensitive to genotoxic stresses that interfere with DNA replication [29, 30]. Deletions or rearrangements at common fragile sites are often observed in many types of cancer, indicating that fragile sites are associated with cancer genomic instability and malignant transformation [31, 32]. It has also been reported that irradiation induces chromosome breakage at fragile sites [19], and the present data was consistent with these previous reports. In particular, we observed deletions harboring *WWOX* located on 16q23.1-q23.2 (FRA16D), in some monoclonal sublines (Supplementary Figure S5B). *WWOX* is known to be a fragile site-associated tumor suppressor gene, and loss or down-regulation of *WWOX* expression has been shown to contribute to the development of cancer [20]. Furthermore, some breakpoints of the chromosomal rearrangements detected in LM200-#25 were located on common fragile sites, including ID23 [2p24.2]: FRA2C, ID30 [2p16.2]: FRA2D, ID80 and ID91 [5p14]: FRA5E, and ID93 [5q15]: FRA5D, suggesting that fragile site aberration by irradiation may contribute to establishment of chromothripsis. Indeed, Kloosterman WP et al. [33] reported breakpoints involved in common fragile site in cases of colorectal cancer with chromothripsis.

The mechanism by which chromothripsis occurs remains unclear, but recent reports indicate that chromothripsis results from rearrangements in micronuclei including one or more chromosomes [10, 11]. This model may be sufficient to explain the characteristics of chromothripsis, i.e., local shattering of the chromosomes. One of the mechanisms by which micronuclei form in the cell is attributed to chromosome segregation errors [34], and also radiation is well known to induce micronuclei *in vitro* [35–37] and *in vivo* [38]. In fact, we confirmed the presence of micronuclei after X-ray irradiation in HOC313-LM cells (Supplementary Figure S2). Although we haven't observed micronuclei after microbeam irradiation using SPICE, micronuclei was shown to be induced by a microbeam with similar radiation quality as SPICE [39]. Therefore, we assumed that the microbeam irradiation by SPICE could produce micronuclei in the HOC313-P and -LM cell lines.

When DNA is damaged by irradiation, ionizing radiation may induce various rearrangements depending on whether the angle of the radiation path relative to the long axis of the chromosome is longitudinal, transverse, or oblique [3]. Moreover, chromosome territory, the arrangement of chromosomes within the nucleus, may also affect chromosomal rearrangements [40]. Thus, we hypothesized that chromosomal rearrangements such as chromothripsis induced by microbeam may be due to local DNA damage and/or locally DNA-damaged micronuclei (Figure 4). In the present study, when SPICE irradiated a 2 μm-diameter circle in the nucleus, chromosomes 2, 5, 7, and 20 may have been just sitting near the irradiated spot, resulting in the occurrence of the chromosomal rearrangements involved in these chromosomes in LM200-#25. Therefore, we believe that irradiation conditions and chromosome territory may be important factors for determining what type of one-off event of chromosomal rearrangements would occur. Finally, our findings may contribute to the clarification of the mechanism of chromothripsis induced in part by irradiation.

## MATERIALS AND METHODS

### Cell culture and establishment of monoclonal sublines

HOC313-P, an OSCC cell line bearing a *TP53* mutation, and its highly metastatic subline HOC313-LM, were maintained in DMEM containing 10% FBS [17]. We believe that these cell lines are genomically unstable, because HOC313-LM acquired a genomic amplification at chromosome 19p13.2 simply through *in vivo* selection, without the need of an inducer of DNA damage. Therefore, we hypothesized that HOC313-LM was likely to harbor a tendency of higher genome instability than HOC313-P and induction of chromothripsis by ionizing irradiation might be possible in HOC313-LM. DSBs in HOC313-P and -LM were induced by SPICE, focused at focal chromosomes in a 2-μm-diameter spot within each nuclei [16]. After irradiation, we picked individual colonies and established a total of 46 monoclonal sublines of HOC313-P and -LM.

### Microbeam irradiation

Cell irradiation experiments were performed by SPICE. All irradiation procedures were performed according to the standard targeting procedures as described previously [16]. Cells cultured in a specially-designed dish were stained with 1 μM Hoechst 33342 (Dojindo) before irradiation and incubated for 30 minutes. Next, medium in the dish was removed and cells were covered with a 6-μm-thick polypropylene film (425, Chemplex Industries, Inc.) to prevent the cells from drying. The dish was then set on the sample stage of the SPICE microscope system to obtain a fluorescent imaging of the cells. Nuclei were irradiated with 100 or 200 protons according to the calculated X and Y coordinates of the nuclei that were extracted automatically by the fluorescent imaging. SPICE focuses 3.37 MeV proton beam (linear energy transfer, LET: 11.7 keV/μm) to spot size of 2 μm in diameter at the targeted cell position, thus the absorbed dose (Gy) of the beam spot area of 3.14 μm^2^ within the targeted nucleus can be estimated as 55 Gy or 110 Gy for the 100 or 200 protons, respectively.

### SNP array analysis

We extracted DNA samples from the HOC313-P and -LM lines and the irradiated HOC313-P and -LM monoclonal sublines. To estimate genomic CNAs in these cell lines, we utilized an SNP array (HumanOmniExpress, Illumina) according to the manufacturer's instruction [41].

### Statistical analysis in SNP array data

We subtracted the copy number of HOC313 or HOC313-LM from that of the irradiated monoclonal sublines and performed statistical analysis by the Partek segmentation algorithm (Ryoka Systems Inc.). We defined the segmentation parameters by: (1) the minimum number of genomic markers, i.e., the minimum number of SNP probe sets to identify a region as a genomic copy number alteration, defined as 30 probes, (2) the *P*-value threshold defined as 0.001, specifying the level of significance that the regions are different, and (3) the signal to noise parameter, i.e., the magnitude of significant regional differences relative to the noise level in each sample, defined as 0.3. In addition, the thresholds for deletion and amplification were set at ≤1.8 and ≥2.6, respectively. To ascertain the validity of these thresholds, we evaluated the genomic copy number status of a thyroid anaplastic carcinoma cell line, 8505C, which has well-known chromothripsis at the short arm of chromosome 9, using these thresholds [3]. We concluded that the thresholds set in the present study were adequate for analyzing SNP data.

### Multiplex-fluorescence in situ hybridization

M-FISH was conducted using a commercially available multicolor probe (D-0125-060-DI, MetaSystems GmbH) following the manufacturer's instructions, with modifications [42]. Fluorescent metaphase images were captured in the AutoCapt mode by using a cytogenetic image scanning system (Axio Imager Z2, Carl Zeiss Microscopy; CoolCube 1, Metafer ver. 4, MetaSystems GmbH) equipped with an appropriate filter set. Karyotype analysis on more than 10 metaphase cells per clone was performed using MetaClient ver. 1.1.1 and Isis ver. 5.4 (MetaSystems GmbH).

### Whole genome sequencing analysis

Mate-paired libraries were generated from 1.1 μg DNA isolated from HOC313-P, HOC313-LM, and the irradiated monoclonal subline, LM200-#25. Mate-pair library preparation was performed as described in the TruSeq DNA PCR-Free LT Sample Preparation Guide Rev.B (Illumina). All cell lines were sequenced using the Illumina HiSeq2500 platform with paired-end reads according to the manufacturer's instructions. Read sequences were mapped by the Burrows-Wheeler Aligner (BWA: v.0.7.12) [43] to the human reference genome (ucsc.hg19.fasta). The removal of the possible PCR duplicate reads was conducted using Picard (http://broadinstitute.github.io/picard) MarkDuplicates, and the conversion of the mapping results into pileup-formatted files was conducted using SAMtools (v.0.1.19) [44]. We entrusted the performance of all WGS processes to Takara Bio Inc., Shiga, Japan.

### Identification of rearrangements

Inconsistent read pairs mapped to within 500 bp of each other were considered to support the same rearrangement. We identified candidate rearrangements in a LM200-#25 sample with >2 support read pairs. Rearrangements that were also observed in HOC313-P or -LM samples were filtered out. To exclude mapping errors, we further performed a BLAST search (ncbi-blast-2.2.30+) of read pairs that supported rearrangements against the reference genome. If a read pair mapped with correct orientation and distance (≤500 bp) with an *E*-value <10^−7^, we excluded the read pair [45]. Reads mapped with more than two mismatches were also discarded. After filtering, candidates supported by >2 read pairs and at least one perfect match pair, were considered as rearrangements (Supplementary Table S2). The orientations of the different mate-pair tags relative to each other in a cluster were indicated as follows: normal sequence was represented as forward and reverse (FR), and abnormal sequences were represented as reverse and forward (RF), forward and forward (FF), and reverse and reverse (RR) i.e., opposite of FF.

### Validation of rearrangements by PCR and Sanger sequencing

To validate the candidate breakpoints of the *de novo* rearrangements detected by WGS analysis, and to identify the correct junction sequences of the rearrangement, PCR primer pairs for each rearrangement were designed to evaluate the rearrangement junction (Supplementary Table S3). Sequences of each PCR product were aligned to the human reference genome (GRCh37/hg19) using BLAST and Genome Browser in the UCSC database.

### Colony formation assay

All X-irradiation were performed using X-ray generator (Faxitron RX-650, Faxitron Bioptics) operating at 130 kVp and 5 mA with a filter of 0.5 mm aluminum at a dose rate of 0.751 Gy/min. To investigate irradiation resistance in the HOC313-P and -LM cell lines, we performed a colony formation assay after X-ray irradiation. We seeded 5000 cells per well of HOC313-P or -LM in a 6-well plate. After 24 hours, cells were irradiated by X-ray at 0 Gy, 2 Gy, and 5 Gy. After one week of incubation, cells were fixed and stained with crystal violet. The stained area was calculated by densitometry using an image analyzer (LAS4000; GE healthcare UK Ltd.), and black area calculation STD software (Gougasha). Differences between HOC 313-P and -LM were tested with the *t*-test and were considered significant at *P*<0.05.

### Immunofluorescence staining

Immunofluorescence staining of micronuclei was performed to confirm their presence after X-ray irradiation in HOC313-LM cells. HOC313-LM were fixed in 2% formalin, permeabilized with 0.2% Triton X-100, treated with blocking solution (1% bovine serum albumin in phosphate-buffered saline), and then incubated with Lamin B antibody (1:100, sc-6216, Santa Cruz Biotechnology) for 1 hour. Alexa Fluor 488-conjugated antibody was utilized as a second antibody (1:100, A-11094, Thermo Scientific). DAPI (1:1000, D1306, Thermo Scientific) and rhodamine phalloidin (1:1000, R415, Thermo Scientific) were used to stain nuclei and F-actin, respectively.

## SUPPLEMENTARY FIGURES AND TABLES








