# Supplementary material for: Chromothripsis-like chromosomal rearrangements induced by ionizing radiation using proton microbeam irradiation system
Source: Oncotarget. 2016 Feb 4;7(9):10182–92. doi: 10.18632/oncotarget.7186 (PMC4891112; doi:10.18632/oncotarget.7186)
Supplement: Supplementary file 1 [file oncotarget-07-10182-s001.pdf]

SUPPLEMENTARY FIGURES AND TABLES

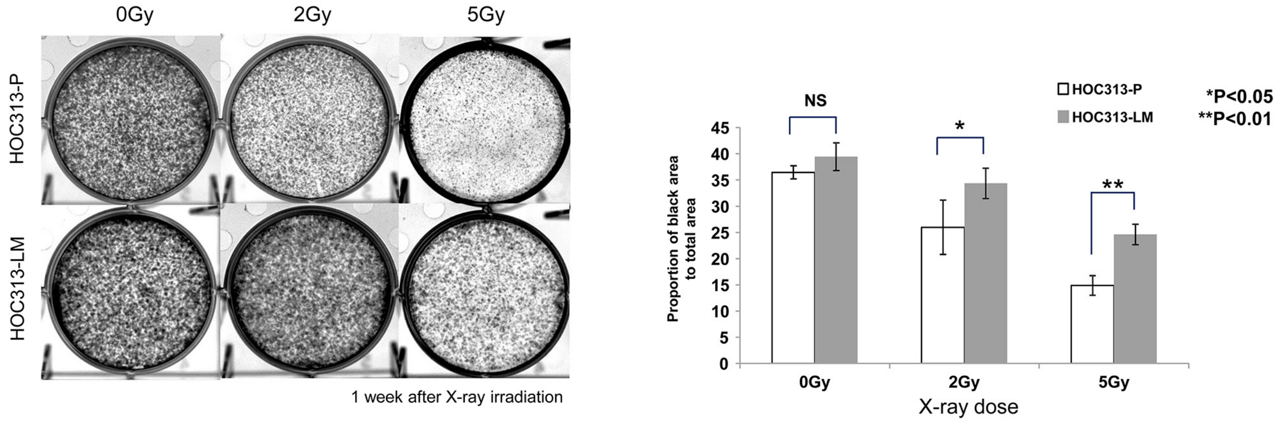

Supplementary Figure S1: Evaluation of radiation tolerance in HOC313-P and -LM. Images of colony formation are at 1 week after X-ray irradiation (0Gy, 2Gy and 5Gy). Bar graph represents the proportion of the black area to the total area. Experiments were performed in triplicate. *: P<0.05, **: P<0.01

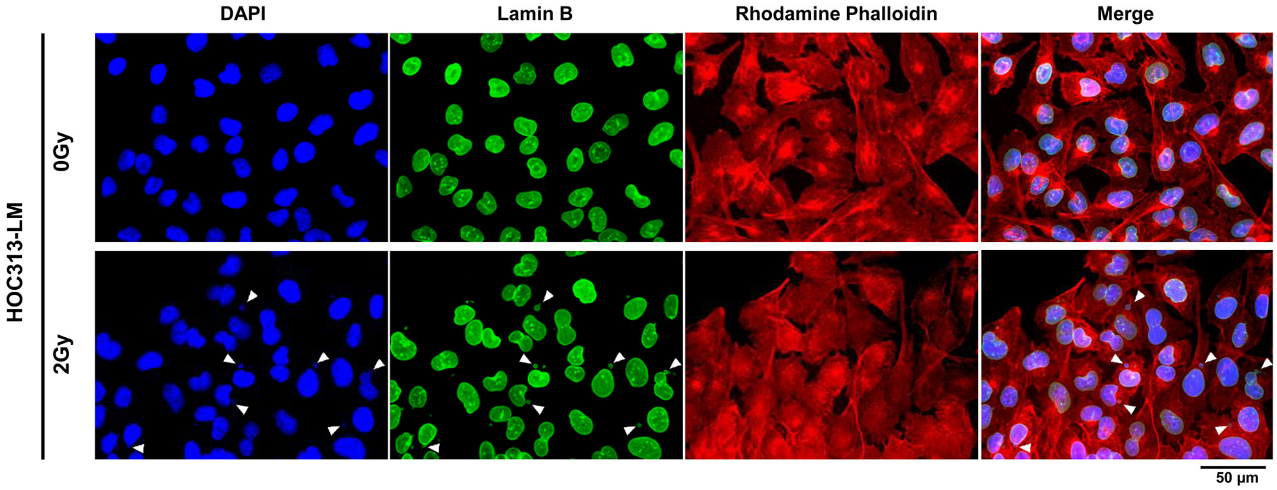

Supplementary Figure S2: The occurrence of micronuclei after X-ray irradiation in HOC313-LM. Immunofluorescent analysis with HOC313-LM at 24 hours post treatment with or without X-ray irradiation. DAPI (blue), Lamin B (green), and rhodamine phalloidin (red). Arrowhead indicates micronuclei.

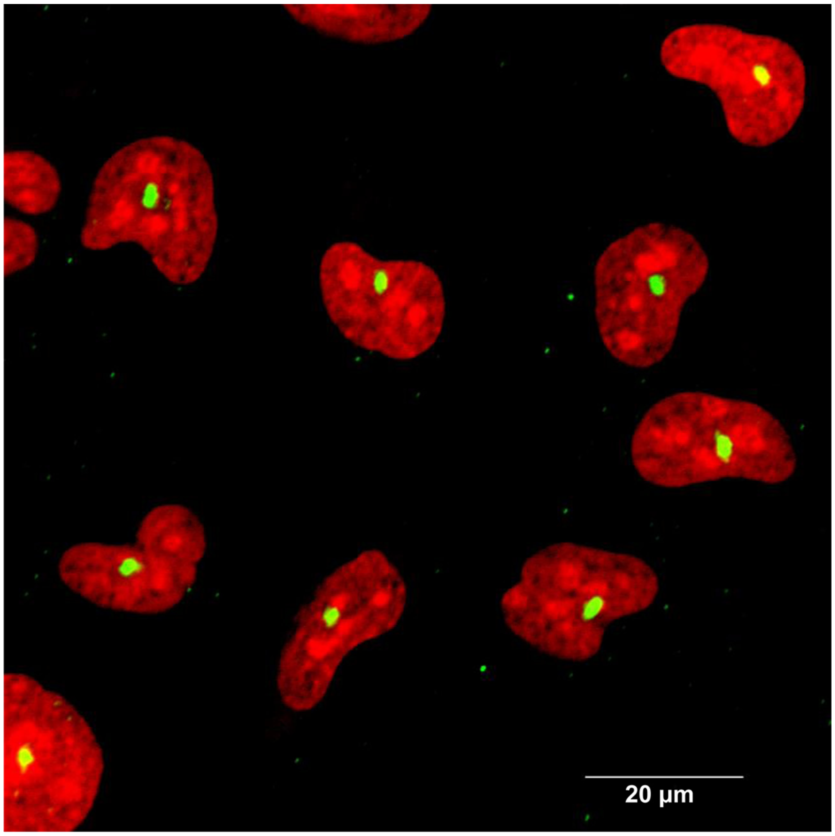

Supplementary Figure S3: Representative image of Nuclei of human lung carcinoma cell line irradiated with SPICE. At irradiated spots of approximately 2 micrometer in diameter, immuno-stained γ-H2AX (green) indicative of microbeam-induced DNA double strand breaks was detected as a green spot in each nucleus. The photo is shown by courtesy of Dr. Teruaki Konishi, a coauthor of this paper, from his original data.

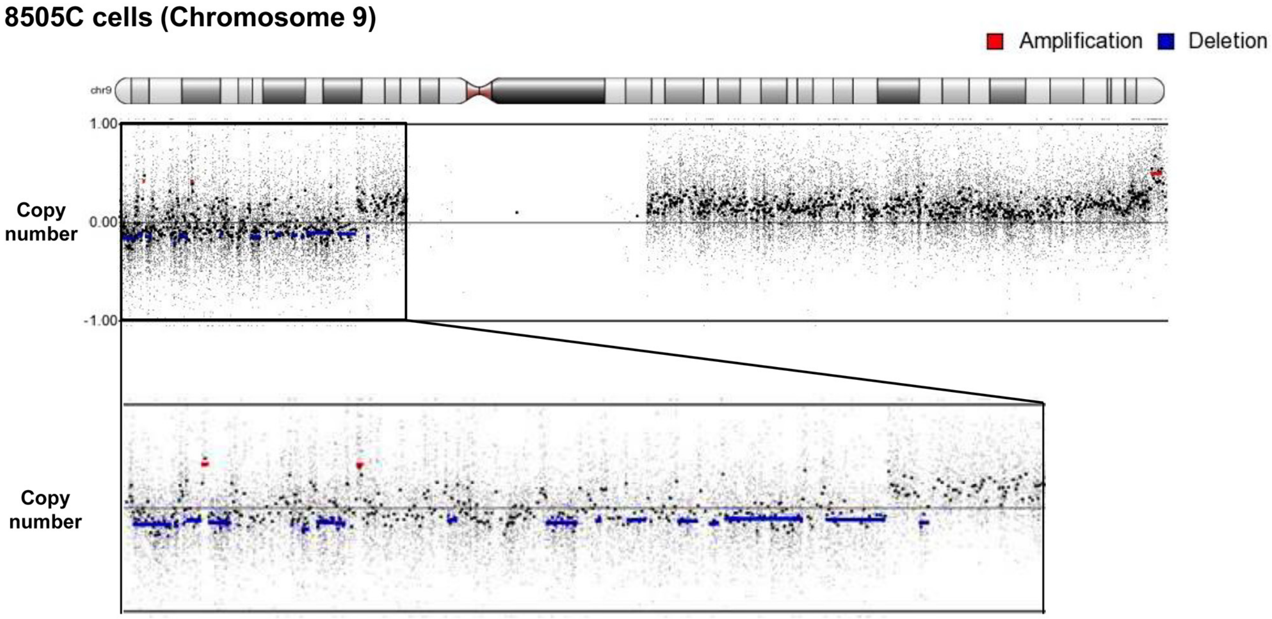

Supplementary Figure S4: The SNP array profile of chromosome 9 in the 8505C cell line after statistical processing as a positive control of chromothripsis. The upper profile is the entire chromosome 9, and the lower is an enlarged view of its short arm. Red dots indicate amplifications and blue dots and lines indicate deletions. The threshold of amplification and deletions were described in Materials and Methods.

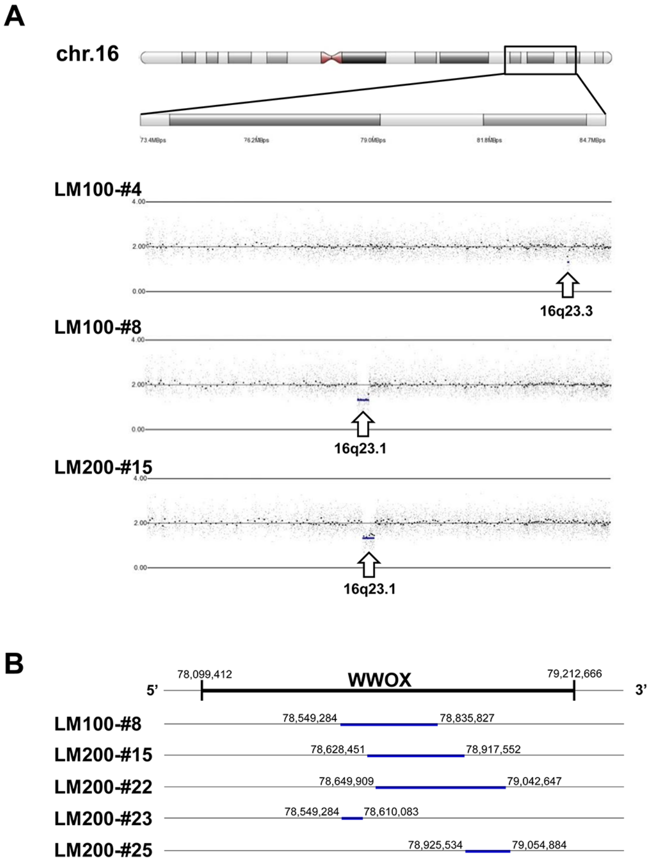

Supplementary Figure S5: Chromosomal deletions around the fragile site FRA16D: 16q23.2 were induced by irradiation at high frequency. A. SNP array profiles between 73.4 Mbps and 84.7 Mbps at chromosome 16 in LM100-#4, -#8, LM200-#15, -#21, -#22, -#23, and -#25. Arrows and blue lines indicate deletions in each monoclonal subline. **B.** Deletion regions in LM100-#8, LM200-#15, -#22, -#23, and -#25 included the WWOX gene, a tumor suppressor gene. The blue lines represent the deletion length of each monoclonal subline.

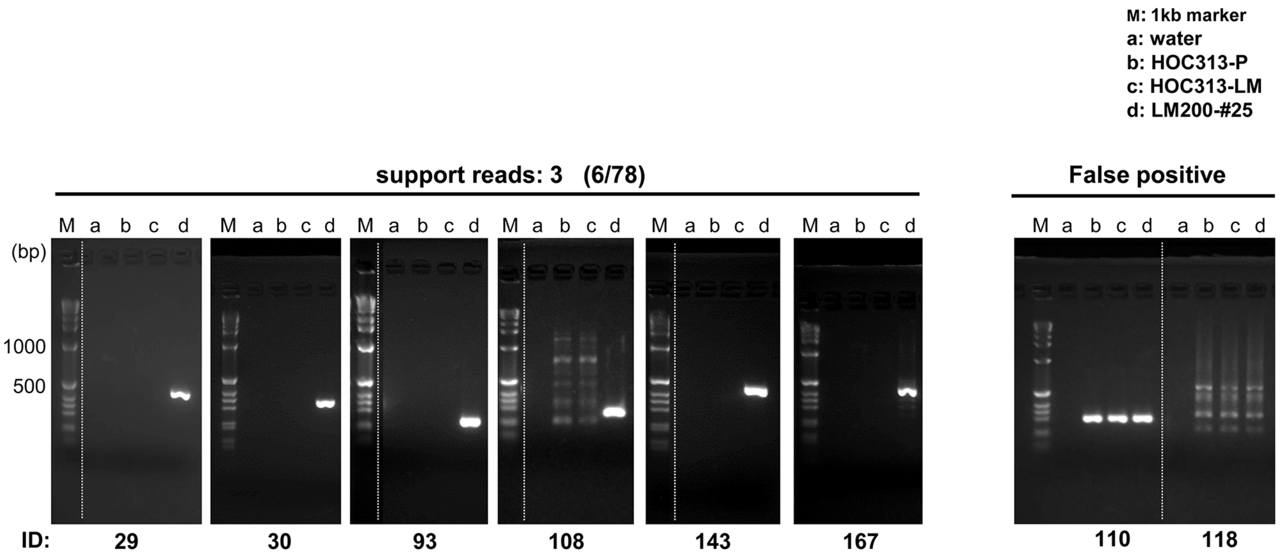

Supplementary Figure S6: Validation of the candidates of de novo rearrangements by PCR. M: 1kb marker, a: control, b: HOC313-P, c: HOC313-LM, d: LM200-#25.

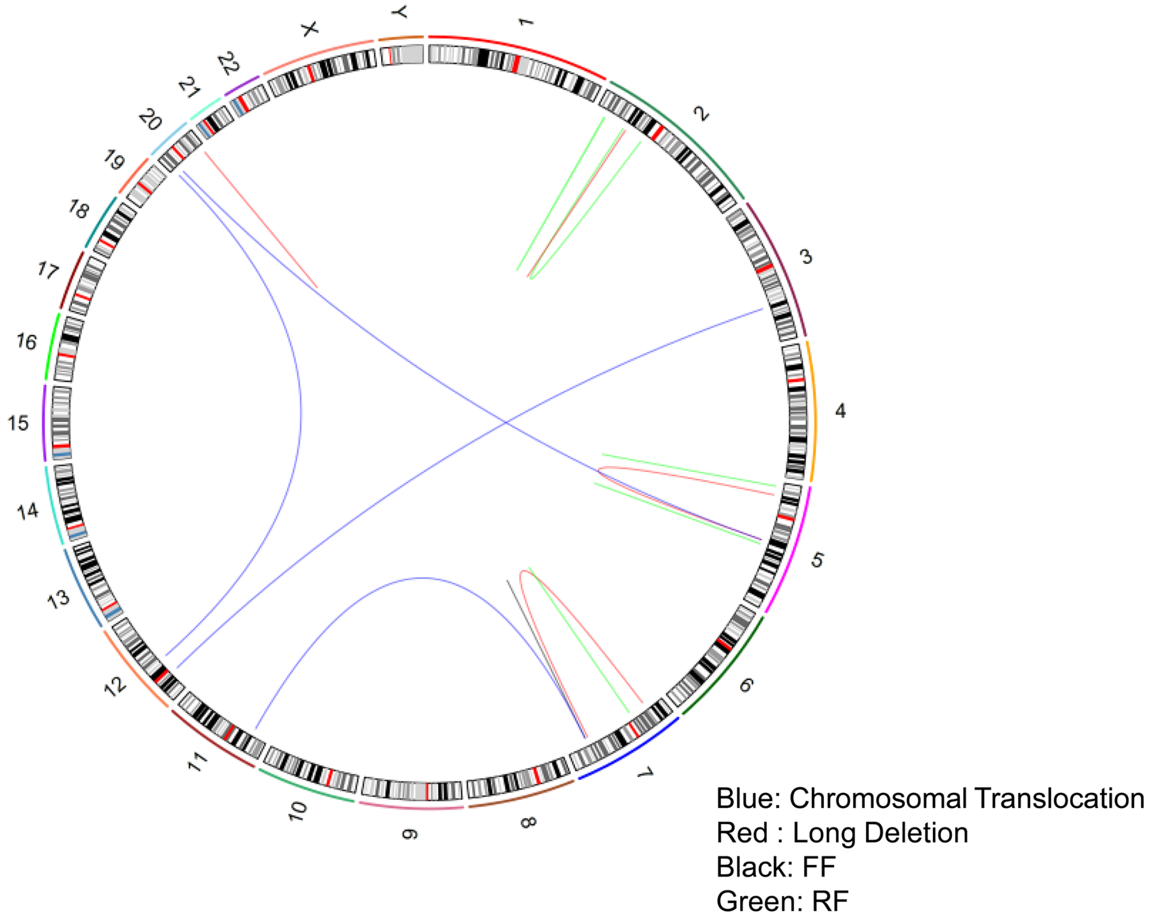

Supplementary Figure S7: CIRCOS plots of LM200-#25 with all chromosomes. Blue: chromosomal translocation, Red: long deletion, Pink: forward and forward (FF), and Black: reverse and forward (RF).

Supplementary Table S1: List of chromosomal alterations in established monoclonal sublines by SNP array analysis

(See Supplementary File 1)

AQ 1 **Supplementary Table S2: List of candidates after filtering of whole genome sequencing data.** The orientations of the different mate-pair tags in a cluster relative to each other are represented as 1 or -1. The correct orientation of a mate-pair tag is represented by 1 (Position 1 orientation) and -1 (Position 2 orientation).

(See Supplementary File 2)

Supplementary Table S3: Sequences of primers for validation PCR

(See Supplementary File 3)

Author Query

AQ:1 Supplementary Table 2 citation missing kindly provide
